# A Practical Treatise on Parturition

**Published:** 1829-01-01

**Authors:** 


### A PRACTICAL TREATISE ON PARTURITION; COMPRISING THE ATTENDANT CIRCUMSTANCES AND DISEASES OF THE PREGNANT AND PUERPERAL STATE. By *Samuel Ashwell.* 8vo, pp. 546. 1828.

THE opposition which *scientific midwifery* has been doomed to struggle with, has now nearly subsided; the natural prejudices of the female, and the uxorious scrupulosities of the male, have gradually given way to the influence of truth; and the united claims of interest and science have ultimately prevailed over ignorance and superstition. That some reluctance would be manifested against the admission of *midwifers* into the parturient chamber was generally anticipated, but this reluctance was expected from the party which have shewn the least; while those whose minds were considered superior to feminine timidity, and whose assent was not supposed to require either much sacrifice of feeling, or much display of heroism, have thrown the greatest obstacles in our way, and have unnecessarily increased that aversion which existed in women against the obstetric interference of men. It may certainly require an effort from the weaker sex to submit, for the first time, to the assistance of our profession, and so far to unsex the delicacy of their nature, as to commit their persons and their lives into the hands of accoucheurs; but every encouragement should be held out which might aid their resolution, and the pardonable prejudices of the wife ought not to be abetted by the whimsical pruderies of the husband.

It must require little discernment to perceive, that the medical practitioner has every advantage over the midwife, which the scientific physician enjoys over the uninitiated empiric, and that he whose resources are sufficient to meet every exigency, must acquire the confidence of his patient sooner, preserve that confidence longer, and be in every respect much more efficiently useful, than one who is generally destitute both of mind and education. The majority of midwives are little superior to the majority of sick-nurses; and although they may conduct ordinary cases safely, where, in fact, interference is improper, the slightest aberration from the common symptoms produces consternation, and, when assistance is required, the case is either abandoned in despair, or transferred to the direction of him, who, when once employed, will seldom afterwards be displaced by the midwife.

Mr. Ashwell's work, which has given rise to these preliminary observations, opens with a hasty sketch of the history of that science, which it is its object to illustrate, and then proceeds, in four parts, to treat of the "Obstetric Properties of the Pelvis;" of the "Gravid Uterus, Conception, Sterility, and the Signs and Diseases of Pregnancy;" of "Parturition, in all its Varieties," and of the "Diseases which belong to the Puerperal State."

The human pelvis may be considered as an arch, on which the weight of the superior part of the body is supported, but both its properties and powers depend much, in different animals, upon its position. Thus, when it is nearly perpendicular, as in the elephant, the greatest degree of power is obtained; when it is nearly horizontal, as in the stag, the greatest share of agility is acquired; and, when a certain degree, both of strength and speed, is required in the same animal, as in the horse, its position is neither horizontal nor perpendicular, but inclined. When the pelvis is well formed, the average measurement of its short diameter, from pubis to sacrum, is four—that of its long diameter, from side to side, five inches; and the diagonal, or a line drawn from the back of the acetabula to the sacro-iliac synchondroses, five inches and one-fourth. But congenital deformity, and many causes operating during life, may so alter these proportions, that the dimensions of the fœtal head and maternal pelvis shall no longer bear any relation to each other. In the greater number of instances, the brim is in fault, both the outlet and intermediate cavity being tolerably natural, and when the contraction of this part is so great as to require instruments, it will generally be found in the short diameter, from an approximation of the ossa pubis to the sacrum. Indeed, Dr. Denman asserts that, when there is any deformity in the superior strait, it will be always found from before backwards. This assertion has, however, met the fate that such general assertions, resulting from mere experience, no matter how extensive, are usually doomed to, for Baudelocque, Dewees, and many others, have had cases, in which the deformity consisted of a contraction of the long diameter. Besides, tumours may form within any portion of the pelvis, and as effectually obstruct the natural passages as original malformation.

But neither deformity of the pelvis, nor tumours within it, are the only causes, on the part of the mother, of instrumental labour; no tumours may exist, and the different diameters of the pelvis may bear a very exact proportion to each other, yet instruments be necessary.

"A *small pelvis* will occasionally give rise to difficulty in parturition; for although it generally happens, if the woman be small, that her child will be small also, and as this kind of pelvis exists apart from contraction, maintaining indeed its shape and symmetry, labour will very frequently proceed with facility. Yet it may so happen, that a small woman shall produce large fœtuses. An instance of this lately occurred to me, where, in consequence of the disproportion existing between the child and the pelvis, I was compelled to use the perforator, after having in vain employed the forceps.

"The treatment of these cases is by no means easy; we are only aware, indeed, of two methods by which, if the deformity be extreme, and the full term of pregnancy be completed, women can be relieved from this most calamitous situation. We may perform embryotomy, or the Cæsarean operation. And, in relation to the first of these proposals, Burns has ascertained that when the standard head is reduced in the best possible way, by the removal of the frontal, parietal and squamous bones, it will require for its passage an aperture of three inches in length, and of one inch and three quarters in breadth. If this space does not exist, the Cæsarian operation, of the success of which we do not in this country entertain any very favourable anticipations, must be resorted to." 69.

To ascertain, with some accuracy, the capacity of the pelvis, several pelvimeters have been recommended, but we question, with the author, whether their indications be much superior to those which may be procured by the experienced inquiry of the finger. If adopted for internal application, their use must be exceedingly equivocal, and, though externally employed, their results must be varied by every variety of figure, and every degree of corpulency.

Referring his reader, for a description of the contents of the pelvis, to general systems of anatomy, the author proceeds, in the *second part*, to consider the laws and diseases of the menstrual function. In this country, menstruation commences about the 14th year, and ceases about the 48th; in Lapland, it seldom begins before the age of 18, and is proportionably later in its stay; while, in Persia, puberty arrives at the age of 10, and Eastern matrons are old women, when ours are merely entering upon the duties of conjugal life. With this variety in the period of its approach, there is as much in the quantity of the discharge, for while, with us, it seldom exceeds six ounces, in the East, 18 or 20 are the ordinary secretion. These varieties are to be accounted for by difference of climate, food, and social habits; a low temperature, and unstimulating diet, being unfavourable to uterine action, while heat and luxury enervate the solids, and increase the quantity of blood.

That the menstrual fluid is nothing but a secretion, is now generally, if not universally, believed, but much obscurity still rests over the physiology of this function, and we can neither satisfactorily explain the causes of menstruation, nor account for the periodicity of its phenomena.

"No part of the anatomical structure of the womb seems to favour the opinion that it may be attributed either to general or uterine ferment; nor are we at all more successful when we look for its cause in universal or local plethora. Were this the case, the quantity of menstrual fluid ought to be influenced by the abstraction of blood from the arm, before or during menstruation, which is not the fact. We were lately particularly impressed with the superior relief of pain afforded only by the commencement of the discharge, to that which we procured by the abstraction of blood in a patient, who was supposed to be suffering under peritoneal inflammation, and from whom under that impression sixteen ounces of blood were abstracted. She had long been the subject of dysmenorrhœa, and in twelve hours after the venesection, the catamenia began to flow, and the cessation of the pain, which was scarcely relieved by the bleeding, was remarkably manifest and decided. Further than this, women in whom no kind of plethora can by possibility be supposed to exist, who, in consequence of dissipation, have impaired their appetites and diminished their strength, yet menstruate regularly. We believe it to be one of the instances in which the researches of able and intelligent men will terminate in their tracing to the will of the Creator; in other words, they will regard it as a law of nature, that the fleshy uterus of the human female, shall, once every month, by a secretory action, produce a certain sanguineous fluid, just as the liver secretes the bile and the kidney the urine. It is true there is the superadded peculiarity of the periodical return. This question will, however, admit of explication when the first part of secretion is understood. If there are some women to whom the catamenia are denied, and such instances are on authentic record, nature almost invariably attempts to remedy the misfortune by setting up some other evacuation, which in a measure supplies the place of the proper one, as far as concerns their health. In some we find a periodical discharge of blood from the nose, from the anus, from the puncta lachrymalia, from the ears, or the nipples, and Baudelocque knew a woman of seven or eight and forty, who, from the age of fifteen, had been regularly attacked every month by a vomiting and purging, which lasted three or four days. She never had the catamenia." 97.

Now, although we are neither prepared nor disposed to enter at present upon this physiological point, we cannot avoid observing that Mr. Ashwell's arguments against plethora being the cause of this discharge appear to us any thing but conclusive. Every practitioner is aware, that in local inflammation we may frequently persevere in abstracting blood from the system until we aneamiate our patient, without subduing the topical disease, and that in periodical head-aches, whose cause has been traced to congestion within, or determination towards the head, we may often use the lancet to syncope without relief, while cupping, to half the quantity upon the nape, or applying leeches to the temples will prove effectual. In the same way, and upon the same principles, the chlorotic girl may be bled to exhaustion from the arm, without calling forth her menses, or removing her disease; and, while strength of pulse, rigidity of fibre, and acute pain, give sufficient indications that there is neither deficiency of strength nor of fluids, leeches to the pubes, or glasses to the loins, will generally effect what more active measures would have failed to produce. There is, therefore, no more force in the objection, that, because the restoration of the catamenia in the case above-mentioned did more for the relief of the patient than the abstraction of 16 ozs. of blood from the arm, the uterus could not have been labouring under any plethora, than were the author to maintain that, because 6 ozs. of blood discharged from the nose during an attack of epistaxis will alleviate head-ache more rapidly than 12 taken from the arm, there could not, therefore, be any congestion within the head; and the occasional occurrence of regular menstruation both to time and quantity, and we would observe, that it is merely occasional, in dissipated females, goes no farther to prove the non-existence of uterine plethora, than the circumstance of a system having been worn down by intemperance could establish the impossibility of any local action, or visceral congestion, ever after taking place. The real state of things, we imagine, to be merely this;—the uterus and uterine system are very copiously supplied with blood from the spermatic and hypogastric arteries;—so soon as the female becomes so matured as to admit of conception, the vascular supply of these organs is increased, and shares in that general development which occurs at puberty;—if impregnation now take place, this *increased* and *excited* circulation finds its own relief in the new action which is set up, and the new demand which is made upon it; but, if not, Nature has furnished herself with a *substitutional outlet*, and a monthly secretion is established, by which, not only the sexual orgasm is moderated, but the uterine economy preserved in a susceptible condition. In advocating this theory of menstruation, we join not issue with such as believe that the menstrual fluid is the fœtal pabulum during pregnancy; the menses, we believe, are not only *visibly* but *essentially* absent during the growth of the child, and the uterine blood, which was expended during the unimpregnated state, in eliminating this secretion, goes, after conception, to answer the demands of the fœtal system. If there be any truth in this view, it must be evident that a general depletion can make no especial impression upon a chlorotic system, and that, so long as the sexual appetite exists, there will be uterine excitement, or, if you will, plethora in a constitution very greatly and very generally dilapidated.

Women are supposed to suffer more than men, at puberty, and it is certain, that this *extra-function* subjects them to many serious affections. Thus, the menstrual discharge may not appear at its proper period, or after its appearance may be suppressed; it may be profuse or scanty, continue too long or be productive of pain. In *amenorrhœa* the author recommends the *ammoniacal injection*, venesection is disapproved of, and the preparations of iron are preferred as tonics. In *menorrhagia*, which he believes, in a majority of cases to arise from hæmorrhages attending early abortions, or some affection of the uterus, the recumbent position, abstinence from mental, medical, and dietetic stimuli, gentle aperients, and cold or astringent injections, constitute our principal treatment. Perhaps, in some instances, venesection might be useful, but only at the very commencement of the complaint, while the strength is unhurt by the discharge, and when we have reason to suspect some vascular activity.

"We must, however, bear in mind, that in cases where the weakness giving rise to menorrhagia is local, not constitutional, we incur some degree of hazard by the employment of general tonic measures, as we are told that by these means we increase the strength of both parts (we suppose he means by *both parts* those that are weakened and those that are strong) at the same time. We confess we are not very fearful on this point, if due care be displayed in the treatment of the local debility: let cold astringent injections be perseveringly thrown into the vagina, at the same time allowing the general health every opportunity to recruit, and we feel assured, that greater benefit will be obtained than if we had carried forward the treatment by a preponderance in favour of local, to the disregard of constitutional remedies." 114.

The interest of the following passage will be a sufficient apology for its introduction:—

"All the common circumstances, Dr. Denman observes, attending menstruation, have been well and fully described by various authors; but having very often observed a substance expelled with the menstrual discharge, which has hitherto escaped notice, and apprehending that the knowledge of this substance might be of use in practice, he felt it incumbent to describe it. 'In the examination of the catamenial discharge, for the purpose of investigating the state of the uterus, and the discovery of some complaints thereon depending, a membranous substance was often shown me, which was usually considered as the token of an early conception, or as the casual form of coagulated blood. But, on examining this substance with more attention, I constantly found that one surface had a flocky appearance, and the other a smooth one; that it had, in all respects, the resemblance of that membrane which Ruysch had called the villous, of the formation of which Harvey had given a very curious description, and which the late Dr. Hunter described with his usual precision, and called the decidua. To put the matter out of doubt, several years ago I requested the favour of Dr. Baillie to examine some portions of this membrane, and he agreed with me in thinking it an organised membrane, similar in structure to the decidua. As the first cases in which this membrane was discharged were those of women who were married, a doubt arose in my mind whether it was not really a consequence of early conception; but I have lately had the most undoubted proofs that it is sometimes discharged by unmarried women, and may be found previously to and without connubial communication; and that the uterus has occasionally, or constantly in some women, the property of forming it, at, or in the interval between the periods of the menstrual discharges. It seems particularly necessary to establish this fact, as the appearance of the membrane has more than once given rise to erroneous opinions and unjust aspersions. Nor is this the only circumstance in which some women, at each period of menstruation, have symptoms like those which accompany pregnancy or parturition. In every case, in which this membrane has been discharged, the women have menstruated with pain, and the discharge has flowed slowly, and apparently with difficulty, till the membrane has come away, which in some cases has been in small flakes, and in others in pieces equal to the extent of half the cavity of the uterus, or more, of which they retain the shape. I suspect, but my experience does not enable me to decide, that this membrane is expelled in every case of habitually painful menstruation.'

"Dr. Denman supposes that no woman can conceive who is affected with this disease; but some cases, says Mr. Burns, and amongst others that related by Morgagni, are against this opinion. *Mercury*, bark, chalybeates, myrrh, and injections, have all been tried without much effect. Time in general removes the disease better than medicine, which is only to be advised for the relief of pain, weakness, or any other symptom which may attend (on) or succeed to this state." 119.

The next chapter our author devotes to a brief description of the gravid uterus, and the following to the subject of conception; but as we are fond of method, even in a Review, which may occasionally lay claim to some logical license, we shall transpose this arrangement, and, preferring the order which Nature herself pursues, take up the doctrine of conception before we investigate the nature of its results.

The difficulties which surround this physiological question have been universally felt and acknowledged, and the different attempts which have been made to remove them have done more for the celebrity of the inquirers than for the elucidation of the subject. Pythagoras supposed that a vapour descended from the brain and nerves of the male during the act of coition, from which similar parts of the embryo were formed, and that the grosser parts were composed of blood and humours within the uterus. Empedocles thought that some portions of the embryo were contained in the semen of the male, and others in that of the female, and that the union of these produced the complete ovum. This opinion was advocated by Hippocrates, and in its leading features does not differ materially from more modern views. Aristotle denied the existence of female semen, maintained that the body of the fœtus was formed of the menstruous secretion, and that the semen of the male imparted to this prepared materialism the principle of life. Galen believed that the embryo was produced by the male, and supported by the female. The views of Harvey, although digested during the researches of many years, are very obscure. Unable to express himself directly, he illustrates the act of impregnation by magnetic phenomena, and tells us that as iron becomes magnetic by being rubbed with a magnet, so the uterus by coition acquires the capability of conceiving. Hamme and Leuwenhoeck asserted that the semen of all male animals contained numerous animalculæ, in each of which were to be found the perfect rudiments of a future animal of the same kind, and that these only required from the female a habitation and nutriment. Objections, however, having been raised against all these theories, the field became open to new adventurers, and Chemistry brought her doctrines of affinity, combination, effervescence, and precipitation, to bear upon the subject; and, although she failed in rendering her views popular according to her hypothesis, the embryo was elaborated with all the precision of her art, and we could as scientifically converse upon the precipitation of a fœtus during the effervescence of the acid male and alkaline female semen, as we could of the chemical combinations and results of purely dead matter.

Leaving these phantoms of unsettled minds, and looking to the results of more enlightened thought, we still meet with much doubt and difference, and the point, which experiment had settled to-day, becomes a subject of dispute after the investigations of to-morrow. Dr. Blundell conceives it demonstrable that, in generation, the female furnishes the rudiments and the male the semen; but whether these two substances must have access to each other for the production of a young animal he does not positively determine. We know that women have conceived from the application of semen to the vulva, the hymen being entire, and Dr. Haighton has shown that corpora lutea have been formed in the ovaria, although the fallopian tubes had been closed previously to sexual intercourse. But Sir E. Home imagines that these bodies are no evidence of impregnation; that they come forward in successive crops during the virgin state, and that they require the actual contact of the male semen to impart to them that fecundity which develops them into life.

After impregnation has taken place the uterine system undergoes an entire change in its functions and economy. The womb becomes altered in its figure, position, and volume. Its blood-vessels increase to a size which has entitled them to the name of sinuses. Cruikshank says its lymphatics become as large as quills, its nerves participate in this development, and the pelvis in the impregnated female may be regarded as the focus of every important sympathy. These very great and serious changes cannot occur without affecting the general health to a certain degree, and producing some disturbance in the general system. Some of them are, however, so uniform in their occurrence, and so intimately connected with utero-gestation, that they merit not the character of disease;—thus, suppression of the menses and enlargement of the mammæ are natural, if not necessary consequences of conception; and others are of so trifling a nature, that they seldom excite attention or require treatment; as cardialgia, nausea, irritability, salivation, tooth-ache, œdema of the lower extremities, costiveness, and piles. But it occasionally happens that more important affections appear, and that the general health is so marred that medical interference becomes indispensible. Retroversion of the uterus, excessive vomiting, and affections of the brain, are among the most important.

The first of these used to be regarded with extreme apprehension, but we know that the timely and continued use of the catheter will often suffice for its removal, and that if even this fail it may continue for some time without creating any serious injury. Our author has seen only one case of complete retroversion, and this was caused by a distended bladder.

"It occurred in the third month, in a patient who, previously to pregnancy, had been the subject of retention of urine. We were compelled to examine, by the severity of the symptoms; the distention of the bladder, and the suffering thereby induced, rendering the lady very solicitous to know precisely her situation. We were not long in ascertaining that the natural position of the os uteri was changed, and after some ineffectual efforts to reach it, we examined by the rectum, and the tumor formed by the fundus uteri, between the intestines and the vagina, was readily felt. The catheter was immediately introduced, and a large quantity of water drawn off. A dose of castor oil, and an injection of simple gruel and salt was advised, and the patient was recommended to lie on the abdomen. The painful symptoms were much relieved, and after a repetition of this treatment several times, things were allowed to pursue their usual course. During the delivery of this lady nothing remarkable occurred." 180.

Since retention of urine is, therefore, the most frequent cause of retroversion, the functions of the bladder should be carefully attended to, especially in delicate females, and such as have been addicted to this misplacement; and should it unfortunately occur, the same means will still be proper, for, as Burns observes, "that the uterus does generally rise spontaneously, if the urine be regularly evacuated, is a fact of which I am fully convinced, from my own experience as well as from the observations of others." If, however, these means do not succeed in replacing it—

"We may then pass one or two fingers of the left hand into the rectum, and by the aid of a piece of sponge, fastened to the end of a small bit of cane, according to the suggestion of Dr. Haighton, the gut may be gently pushed up, until it comes in contact with the promontory of the sacrum, thus making pressure on the retroverted womb: two fingers of the right hand may now be advantageously introduced into the vagina, so as to depress the cervix uteri, thus acting on the womb from two points. If we fail after a judicious repetition of these measures, Dr. Hunter advises that the ovum should be tapped, so that by letting off the water the bulk should be diminished. This will very often be attended with great difficulty, and if we succeed the bladder will require very sedulous attention; as, till the *quickening* is fully established, we must not allow any extensive accumulations of urine in this viscus. It is scarcely necessary to remark, that when the uterus has fairly emerged from the pelvis into the abdominal cavity, it cannot again be retroverted." 184.

Cases of severe vomiting are occasionally met with, in which food as well as medicine is rejected, and when the strength must be maintained by nutritious injections. Small doses of calcined magnesia or soda water, taken two or three times daily, leeches and plasters of cantharides or opium to the epigastrium, lemon juice and the mineral acids, when alkalies are ineffectual, and brandy in very small quantities frequently repeated, have been recommended, and may be tried according to the circumstances of each case.

"If, notwithstanding every remedy, the vomiting goes on to debilitate the patient, she may be reduced to a state of extreme danger; in these circumstances, *after consultation*, we think it very justifiable to induce *premature* labour. Dr. Marshall Hall gave us the particulars of a case, occurring under his own notice, although not under his own care, where vomiting continued in spite of every remedy which a most experienced practitioner could suggest, and which terminated fatally in the seventh month. Here premature labour would probably have saved the patient." 194.

Slight cerebral disturbance is not an unusual attendant upon pregnancy, and whether it proceed from topical plethora or irritation, the most careful, and, in some instances, the most prompt treatment is required. Vertigo and headache are often the fore-runners of convulsions, and if such warning symptoms be neglected, the practitioner may be doomed to witness scenes over which he has allowed himself no control.

Besides these disorders, which may be traced to utero-gestation, there are others of a no less dangerous nature, with which it may be complicated without having any etiological relation. Thus, dropsy and syphilis may occur during pregnancy, and place the patient in circumstances of the most imminent danger. In a case of dropsy, so long as the fluid remains within certain limits, and does not, by excessive accumulation, endanger delivery, our treatment may be palliative; but if, by its distention, it occasion pain, and put the life of the patient into hazard, premature labour must be induced, or the operation of tapping performed. In an interesting case by Mr. Langstaff, where, from the size of the abdomen, advanced state of the disease, and debilitated condition of the patient, Drs. Farre and Davis decided upon the propriety of inducing premature labour, the liquor amnii was discharged; but as no signs of labour appeared upon the following day, and the powers were sinking, tapping was carefully performed; 25 pints were drawn off in the course of three days, labour pains came on, 50 ounces of blood being taken during the interval between the delivery and operation, and in 10 days after the labour the patient was considered out of danger. Encouraged by the success of this case, he would recommend the trocar before the hydropic symptoms became severe, and would trust to the lancet for the prevention of any fresh accumulation. But it is obvious, that such cases of dropsy must be the result of simple inflammatory action, and that, where effusion depended upon diseased viscera, this treatment would prove less fortunate.

"*Syphilis* is occasionally complicated with pregnancy, and there is no doubt of the perfect safety of submitting a patient, under such circumstances, to a judicious and mild mercurial course. By this treatment the mother will be cured, but we are not always so successful with the child; for, although it may be born free from any appearance of disease, yet in the course of a few weeks, symptoms of syphilis are not unfrequently occurring. In a lady we lately treated for this disease, and who became pregnant during its continuance, the child was born with copper-coloured spots all over its body, although the mother had been perfectly cured for at least five months previously to delivery. This infant was subsequently affected by thrush, and ulcers soon appeared on the arms and labia pudendi. At this time, we were not in attendance, but we were informed by the gentleman who had charge of the case, that these sores resisted all the simple methods of treatment, and did not heal *till a mercurial plan was pursued.* Mr. Hey, whose reputation has scarcely been excelled by any surgeon in this country, states '*that syphilis, in its secondary form, may be communicated by the mother to the fœtus in utero. That the fœtus may be contaminated when the mother has never been previously affected with lues venerea in the organs of generation,* and when *no apparent disease* existed in the father at the time of impregnation.' 'That *the mother remaining in health shall produce several children, each of which shall in succession become affected with syphilis a few weeks after its birth;* and that the disease shall assume a *milder form, in each child comparatively,* until it be finally worn out.' Mr. Hey confirms these separate statements, by a reference to clearly defined cases, in the treatment of which he completely succeeded by the mercurial plan." 207.

The term of utero-gestation in the human female has been differently calculated, and varies in duration, from the different periods at which these calculations are commenced. Some begin immediately after, while others reckon from a fortnight subsequently to the last appearance of the catamenia. Our author believes that few cases will be found to exceed forty weeks; Hippocrates extended the term to ten, and Haller has adduced cases occupying twelve months. The Roman law allows ten months to elapse between the death of the husband, and the birth of legitimate offspring; France has adopted the same period; but, in England, no time is specified, preferring to leave disputed cases, as they occur, to be investigated by those who are competent to estimate the importance of every accompanying circumstance, and to apply every new light, which the advancing state of midwifery may elicit, to the peculiar exigencies of individual instances.

Mr. Ashwell arranges all labours into three classes—*natural*, *difficult*, and *flooding* labours. In *natural* labour, the head of the child presents at the full time, and the process is completed within 24 hours, without artificial aid, or the occurrence of any morbid affection; it may be *quick*, *lingering*, or *twin labour.* In *difficult* labour, any part of the child may present, and the natural powers are generally insufficient to accomplish delivery. This class contains three orders;—1st, Presentation of the breech, of the extremities, or of any combination of these, requiring manual aid;—2dly, Labours requiring instrumental aid, which can either save both mother and child, or preserve the mother at the expense of the child;—3dly, Impracticable labour, in which the child cannot pass through the pelvis, even with the aid of instruments, and where the Cæsarean operation becomes necessary. In *flooding* labours the author includes the earlier and later hæmorrhages attendant on gestation as well as parturition. Dr. Smellie supposes that out of 1000 cases, eight shall be instrumental or require turning; in two, the superior extremities will present; in five the breech; in two, the face; in one, the ear, and, in ten, the forehead shall be turned to the acetabulum. Dr. Bland says, that of 1897 cases, 1792 were natural, 63 were unnatural; 17 were laborious, and 9 had uterine hæmorrhage before or during labour. But such registers can only be depended on to a certain extent, since some may, from accidental circumstances, treat much more than their proportion of difficult cases. Thus, Dr. Hagen, of Berlin, says, that in 350 deliveries, 93 required the forceps, 28 the crotchet, and 20 of his patients perished; while Dr.

Dewees, of Philadelphia, asserts that, out of more than 3,000 cases, he has not had one requiring the crotchet.

"Many hypotheses have been proposed to explain the phenomenon why labour should so unvaryingly occur at the expiration of forty weeks? Into these we shall not enter, as we know scarcely any thing of the circumstances which exist between the uterus and the child, rendering the contraction of the former necessary at this period. The most natural supposition would ascribe to the development of the fœtus the necessity for this action, but yet we are informed, that in cases of extra-uterine conception, at the expiration of nine months, the uterus is thrown into contraction, similar to that which is produced by the residence of the fœtus within its cavity. It is, however, true, that at the expiration of the above-mentioned period, changes occur, which intimate pretty clearly, that some important process is about to be commenced; and these changes have been denominated the predisposing signs of labour. There is at this period, *a partial subsidence of the abdominal enlargement*, arising from the forcing *downwards* of the uterine contents, by the *contraction* of the fundus; and many women are able correctly to foretel, from this circumstance alone, the early occurrence of labour. Whenever the fundus of the uterus is thus descending, it indicates a favourable disposition to parturient action, and perhaps a good formation of the pelvis; for if, after the commencement of labour, the patient complains that the child is very high, it is unfavourable, indicating perhaps, either that the *fundus* is *inactive*, disposed to act *irregularly*, or that *some pelvic obstacle* may exist. Patients, who have previously borne children, often predict with great accuracy, the early or late coming on of labour, from the occurrence of circumstances and sensations which are unintelligible to any other individuals.

"The follicular apparatus about the neck of the womb is now pouring out a *glairy* and *viscid* secretion; which, when it immediately precedes labour, and from the rupture of some small vessels is of a sanguineous colour, we denominate *shew*. Some women, from their manner of walking at this period, and from the statement of their feelings about the pelvis, would induce the belief, that there was some relaxation of ligaments, and that the popular expression of falling in pieces, is not altogether unjustifiable. These changes are often accompanied *by irritation of the bladder*, and *by griping tenesmic sensations* in the intestines and rectum. These are the principal circumstances inducing the belief, that the expulsatory action of the uterus is about to commence; and, in the language of old Avicenna, that the "appointed time having arrived, labour comes on by the command of God.'" 227.

Passing over the author's history of a quick natural labour, as containing nothing of particular interest, he proceeds to observe, that, from defective expulsory power in the mother, or increased resistance to the exit of the child, more than 24 hours may be consumed by delivery, and the labour may be rendered tedious, or *lingering*. In tedious confinements, depending upon the former cause, the patient's strength must be supported by mild nourishment, the bowels preserved soluble by gentle aperients, and a few drops of laudanum may be given, if the pains harrass, without expediting the labour. Some have strongly recommended active stimuli, and, although unfriendly to their early use, our author acknowledges their necessity under specified circumstances. If the os uteri be dilated, and the head stationary for some hours, if the pains be weak and ineffective, and the means already alluded to have failed in giving energy to the contraction of the uterus, we may allow some wine-negus and solid nourishment, or have recourse to the secale cornutum. This medicine has been considered very destructive to the life of the child, but, from what we have seen of its effects, we are disposed to regard death, when it has occurred, as arising from its too early administration, or some other abuse in its application. If the position of the child be unfavourable, or the os tincæ rigidly close, it is evident that the use of a stimulus, whose specific action is upon the utrine system, must be injurious, if not destructive. It may sometimes produce no palpable effect, in common with other medicines; but, if employed in appropriate cases, and at judicious periods, the operation is generally rapid and salutary, neither inducing hæmorrhage, nor other disagreeable results. Dr. Bibby of New York, asserts, that when the fœtus has been for some time dead, the ergot is altogether inert, so that, supposing this assertion to be correct, which we strongly question, it appears to exert some influence over the fœtal system, while alive in utero. But, where the cause of delay exists not in debility but strength, not in weakness of the expulsory power, but in increased resistance, as from rigidity of the os uteri and external parts, an opposite treatment will be required. The rectum and bladder must be emptied, the patient kept cool and on low diet, and blood should be abstracted. Opiate injections have been advised, and Dr. Conquest recommends friction with belladonna.

"Some time since, I attended Mrs. ———, aged twenty-two, in her first confinement, the edges of the os uteri being much attenuated, very hot, not at all moist, nor disposed to dilatation. There was neither fulness of pulse nor heat of surface, beyond what commonly occurs in the commencement of labour. The bowels were fully relieved by castor-oil, and the pains had been tranquillised by thirty minims of the tincture of opium. After an hour's sleep, she awoke refreshed, and the pains recurred. After the lapse of eighteen hours, there was no alteration in the os uteri. A drachm of the extract of belladonna was rubbed about the cervix and os uteri, but, after waiting for two or three hours, there was no perceptible effect. The pains were very vehement and forcing, but the attenuated edges and heated condition of the os uteri still continued, and it was not dilated more than enough to admit the tip of the finger. A clyster, *composed of six ounces of linseed tea and sixty minims of the tincture of opium*, was thrown into the rectum: it remained nearly an hour, and immediately on its expulsion *two* grains of solid opium, softened by mucilage, were insinuated into the bowel, just above the sphincter ani; very shortly afterwards, the condition of the os uteri began to change, and, before the expiration of three hours from the administration of the injection, the head was protruding at the outlet. We have frequently, since this period, adopted the practice of giving opiate clysters and suppositories, and always with decided advantage." 275.

Mr. Burns, and the generality of other systematic writers upon midwifery, have devoted a distinct class and a separate chapter to labours complicated with anomalous accidents, as hæmorrhage, syncope, or convulsions; but the present writer has abandoned that arrangement, because all these occurrences may happen when the presentation is natural, and subjoins some account of them to his history of *natural* labours. These affections are arranged into two classes —such as are remediable by proper treatment, and such as are replete with danger from the moment of their appearance. To the first belong obesity, syncope, rigors, vomiting, fever, hæmorrhage, obliquities of the uterus, distended or prolapsed bladder, prolapsus ani, œdema of the cervix uteri and external parts, mal-positions of the head, and descent of the vagina and bladder, tumours in the pelvis, and convulsions. We shall confine our attention to two or three of the most important.

*Mal-positions of the head* may produce much difficulty, if not rectified in proper time. The safest and most natural presentation is that of the vertex, but even here derangement may occur, as the long diameter of the head may be opposed to the short diameter of the pelvis. Of this deviation there are two kinds;—where the vertex is found behind the symphysis pubis, and where the forehead, or anterior fontanelle occupies the same position. In the former case, if the pelvis be well shaped, and the head of ordinary size, delivery may be effected without any interference, but if not, the hand must be introduced into the vagina, and the vertex turned to one of the acetabula. Of face presentations we may have four varieties, "first, where the child (chin) is opposed to the pubes," when, if there be no mal-formation in the pelvis or head vigorous pain will generally suffice for the termination of the labour, but if any disproportion exist between them, turning may be attempted, and the feet made the presenting part. The author prefers, however, introducing the hand into the uterus, placing the fingers over the vertex and the thumb upon the centre of the forehead, and during the absence of pain pressing the head and chin upwards and laterally, while, by the fingers he depresses the vertex, and places it against the sacro-iliac symphysis. If this form of presentation be discovered early, the lever may be preferable to the hand. It must be introduced by the side of the pelvis, and passing over the vertex obtain a firm bearing upon this part. When, however, the face is low down and firmly jammed, the pains weak, the parts hot and tumid, and the descent of the head scarcely perceptible, the forceps become necessary, the blades passing over the cheeks to the occipital part of the head, and the locking being effected at the lower part of the face. In the second and third varieties of face presentation, where the chin is opposed to the right or left side of the pelvis, the same treatment is applicable; and in the fourth variety, which is extremely rare, when the chin is opposed to the sacrum, turning should be performed if the os uteri be sufficiently dilated, soon after the rupture of the membranes. But the child is seldom saved under this presentation, and if turning prove impracticable, and the forceps unsuccessful, the author thinks we should prevent any further risk to the mother by opening the child's head.

*Lacerations of the uterus, vagina, and bladder* are distressing accidents, which admit of little relief from professional skill, and have been often left, even by eminent men, to the resources of nature. A variety of causes have been assigned, as the struggles of the fœtus, mental emotion, thinness of the uterine parietes, and their mechanical perforation by pressure and attrition; but the more probable are, external injury and internal mischief, from excessive contraction, awkward employment of instruments, mal-formation and tumours. Dr. Denman has observed, that more women have recovered under such circumstances, who have not been delivered, than those who have, and advises the patient to be resigned to her natural resources; but Mr. Ashwell very properly impugns this principle, and recommends delivery. If the uterus be ruptured, and the child have partly or entirely escaped into the abdominal cavity, the hand should be passed into the abdomen, and the child extracted by the feet; but if, at the time of laceration, its head be engaged in the pelvis, the forceps will generally be required. If the bladder have been lacerated, and the patient survive the first symptoms, the cure will mainly depend upon the catheter, and upon keeping the edges of the wound as much as possible in a state of apposition. A successful and interesting case of this description is given by Mr. Guthrie, of Kilmarnock, in which a piece of sponge was introduced into the vagina, and preserved over the rent in the bladder, and a catheter was fixed in the urethra to prevent the urine from accumulating, by which simple means the aperture became cicatrized in a month, and the patient, upon being examined five months afterwards, was found perfectly free from complaint.

*Convulsions* may occur during gestation, at the time of labour, or after delivery; but as they seem to arise from the same cause at all these periods, they require very similar treatment. They are usually ushered in with singing in the ears, sense of weight about the forehead, and indistinct vision. The pulse is strong, slow, and labouring, there is great inclination to sleep, much sighing, and pain of head frequently alternates with uneasiness of stomach. The following is a good specimen of this disease, and will serve to illustrate the most efficient mode of treatment.

Mrs. A. æt. 18, was slightly indisposed during her first pregnancy, at the end of January, for which, 10 ozs. of blood were taken from her arm. About a fortnight after slight diorsal pains came on, and she was seized with convulsions. Twenty ozs. were taken in the first instance, but the fit returning in the evening, 24 more were abstracted, and an enema was given; at 5 o'clock she was again convulsed, the bleeding was repeated to 10 ozs., and a smart purgative was exhibited. This depletion had no effect upon the os uteri, which was found not at all dilated, though the pains were forcing and strong. The head was shaved, and a cold lotion applied, 30 drops of laudanum were taken, and the nape was blistered. During the morning of the next day she became delirious, slight tremors were observed, and the countenance was pale, but in the afternoon she rallied, and at night the pains became regular, the os tincæ opened, and she was safely delivered of a healthy boy. In two days from this period she had entirely recovered.

"We wish again to urge upon the practitioner the extreme importance of *early and large bleeding* in convulsions during labour, the quantity, not being so much regulated by the number of ounces, as by the effect produced, and it must also be remembered, that there is rarely any safety for the patient, till the delivery is accomplished. If the os uteri be fully dilated and the external parts free from rigidity, the *ergot* may be advantageously used; indeed, Dr. Waterhouse, of Philadelphia, affirms, that this remedy alone is most efficacious in the cure of puerperal convulsions. If, under the above circumstances, the symptoms are very urgent, and the child is still at the brim, turning may be attempted, or if from its producing a recurrence of the convulsions, this measure be deemed unadvisable, we may use *the long forceps,* the perforator being reserved as a "dernier resort" in extreme cases. Again, if the head be low down in the pelvis, and the convulsions are increasing in severity, we can have no hesitation to expedite the birth by the employment of instrumental aid." 330.

*Difficult Labours.* When the inferior extremities present, the patient requires great attention, as much of the facility of delivery will depend on the period when assistance is afforded. The os uteri, being deprived of the pressure of the head, the first stage generally occupies a long time; but so soon as the nates are born, the period for assistance has arrived. The author, having merely introduced this subject, very abruptly runs into a description of the operation of turning, in shoulder presentations, and into Denman's and Douglas' views of spontaneous evolution, imagining, we suppose, that he should consider under the head of Inferior Extremity Presentations, those forms of labour which are *turned* into this order. But it is an abuse both of language and method to treat of cases *naturally* presenting by the feet, and cases *forcibly formed* into footling presentations, in the same section. The operation of turning is of great importance, but should be described in connexion with such presentations as require it, and not with such as are footlings naturally. Indeed, the arrangement of his chapter upon difficult labours is remarkably defective. In the first paragraph of the first section he professes to treat of presentations of the inferior extremities, but, excepting two or three preliminary remarks, devotes it to turning, spontaneous evolution, and embryotomy; in the third paragraph of the same section, presentations of the feet are considered, as though they were distinct from inferior extremity presentations, and the second, third, and fourth sections, which are set apart to the consideration of instrumental labour, are headed by the names of obstetrical instruments, inducing any common reader to expect a description of the instrument, rather than of the cases in which it is required, or of the manner in which it is to be employed.

If the toes present to the symphysis pubis the head is unfavourably situated, and, grasping the extremities, we must, during a pain, give such an inclination to the child as shall direct its abdomen to the mother's spine. Some leave the arms to come in company with the head, but this principle is objectionable, because much difficulty may thus be given to their passage, if the pelvis be small. When, therefore, the axillæ of the child are upon a level with the external part of the mother, two or more fingers should be passed up to the elbow, when the arm may be brought down over the face, and having accomplished the birth of one the second is extracted with ease, and thus is the passage of the head facilitated. If any difficulty should arise, assistance must be speedily afforded to prevent compression of the cord; for this purpose, passing two or three fingers of the right hand over the child's nape, and insinuating a finger of the left into its mouth, we depress the chin, and moderately bring forward the head.

In presentations of the breech the labours must not be interfered with, until the nates have protruded through the external parts, when the case should be treated as a footling presentation.

When the superior extremities present, the position of the child requires alteration before delivery can be effected; or, in other words, the operation of turning is necessary. On this point all agree, but there is great diversity of opinion as to the precise time when it ought to be undertaken. If the membranes be entire, the os uteri dilated, and the hand protruding, our course is obviously to introduce our hand into the uterus during an interval from pain, rupture the membranes, reach the feet, and so bring down the body, that one ear may be towards the sacrum, and the other behind the symphisis pubis.

"*Where the waters have been long evacuated* before the practitioner sees his patient, *the os uteri not fully dilated*, and the pains *vehement and quick*, turning must not be attempted. We are aware that some practitioners do not concur in this opinion, urging that the uterus will most probably be ruptured by the continuance of the pains, if the child remain in this untoward position. The danger of rupture from delay, cannot exceed the risk of laceration, were the hand, for the purpose of turning, to be introduced into a uterus so powerfully contracting. In these cases, and I have seen several, I bleed the patient, if at all plethoric, to fourteen or twenty ounces, and follow up the abstraction, by sixty or eighty drops of the tinct. opii; by these means the uterine action will most probably be much diminished, and then the turning may be safely attempted. It is urged that, although the uterus be not ruptured, yet the continuance of the pain will force the arm, the shoulder, or perhaps the head of the child so firmly into the uterus as to render it impossible to turn, even when its inordinate action has ceased. We are quite alive to the difficulty and risk which attends this jamming of the fœtus into the pelvis; but we think it less than what would accrue from thrusting the hand into this viscus when rigid, powerfully contracting and irritable. We have seen one case where attempts had been made *during the contractile efforts*, to rectify the position of the child. Opium alone had been given, without any previous abstraction of blood. Several attempts proved unsuccessful, and eighty drops of laudanum were exhibited, the woman being undisturbed for some time; after this period the child was extracted with ease, but the uterus had ruptured, and she died a few hours after delivery." 357.

Cases do occasionally occur where Nature is unable to relieve herself, either from weakness in her expulsive powers, or from the unusual resistance she is called upon to overcome. When the pains are so weak as to make no impression upon the head, or have ceased at the end of the second or third day, if the pulse and countenance be expressive of exhaustion, and if we have head-ache, mental inquietude, furred tongue, hot skin, vomiting, and abdominal tenderness, we may rest assured that our patient has approached to a state, from the danger of which instrumental aid will alone deliver her. The instruments now in general use are the long forceps, short forceps, vectis, and blunt hook; perforator, craniotomy-forceps, crotchet, and scalpel; the last four of which sacrifice the life of the child to the safety of its mother. The fang forceps are peculiarly applicable to those deformities of the brim of the pelvis which are produced by contraction of its antero-posterior diameter, and, in the author's opinion, have a decided advantage over the lever, for when once introduced they can scarcely fail to accomplish delivery. They are likewise highly serviceable in those cases of hæmorrhage, syncope, or convulsions, which arise before the head has descended so low into the pelvis as to be within reach of the common instrument. The short forceps can only aid delivery, when the head has decended into the pelvis, the criterion of the propriety of their application being a capability to feel one or both ears. They may be required in three varieties of vertex cases; first, where the turn is completed, the face lying in the hollow of the sacrum; secondly, where the turn is completed, but the forehead is opposed to the pubes; and, thirdly, where the turn is completed the occiput lying to the one, and the face to the opposite side of the pelvis. When the head, from its extraordinary bulk, or the pelvis, from its distortion, mechanically prevents the birth of the child, those instruments, which are employed in embryotomy, will be required. The apparatus of screws and hooks, pincers and perforators, which were in use among the ancients for the performance of this operation, was as complicated as it was dangerous, and the practice of diminishing the head by emptying the cranium is a modern improvement, which has rendered the operation less dangerous by making it more simple. If the perforator be fully employed, very few instances can occur in which delivery may not be effected through the natural passage, but it occasionally happens that the capacity of the pelvis has been so encroached upon by malformation or disease, that even the destruction of the child cannot purchase the safety of the mother, but both must be subjected to a most hazardous *experiment*, to furnish either with a chance of life. Of 22 cases, in which the Cæsarian operation has been performed in these islands, 21 of the mothers perished, 10 of the children were still-born, and only 4 of the 12, who were extracted alive, survived for a few days. To diminish this sacrifice of life several proposals have been made, as the section of the symphysis pubis, abstinence, and the induction of premature labour. The first proposal has passed into disuse, and the second cannot be relied upon; for women, who reject by vomiting every particle of nourishment, have as large children as others. Of the third plan most practitioners think highly; and, although the mother incurs some degree of danger, she is placed in as much, perhaps, by the use of the perforator. Out of 33 cases of labour prematurely induced in the 8th month, as given by Dr. Merriman, 21 children were born dead, four lived a few hours only, and nine survived. Thus, nearly one-third of the children were saved, who must have been destroyed by the perforator, had their mother gone to the full time; and all the women recovered, the majority, if not the whole, of whom must have perished, had the Cæsarian operation been performed.

*Flooding Labour.* In the early months of pregnancy, comparatively little blood is sent to the uterus, and, therefore, the floodings which occur during this period are seldom dangerous, unless the placenta become extensively detached; but when utero-gestation has far advanced, the uterine vessels enlarge, the blood which they circulate becomes very much increased, and the liabilities to hæmorrhage are proportionably multiplied. Our author considers hæmorrhage under four divisions, with relation to the period of its occurrence.

"*First.* Hæmorrhage occurring *previously to the completion of the sixth month,* which may or may not be accompanied by abortion, and which rarely requires manual aid.

"*Second.* Hæmorrhage occurring after this period, but *before the birth of the child,* including partial and entire presentations of the placenta.

"*Third.* Hæmorrhage occurring *subsequently to the birth of the child* and before the expulsion of the placenta.

"*Fourth.* Hæmorrhage occurring *after* the expulsion of the placenta." 410.

Cases of the first kind are dangerous, in proportion to the age of the pregnancy, the extent of placental detachment, and the sudden, or more gradual, escape of the blood. If the patient have a good constitution, and be plethoric, bleeding to twelve ounces, mental and corporeal quiet, gentle saline aperients, and mild enemas, will form our principal treatment; but if the patient have lost much blood in the first instance, if the hæmorrhage be frequently recurring, and the strength much exhausted, a state of collapse may be feared, and more cautious management is called for. If it can be safely done, the strength should be permitted to rally without the aid of any artificial restorative; but, if not, some diffusible stimulus may be exhibited, in very small and repeated quantities, and absolute rest, large doses of digitalis, small doses of superacetate of lead, and ol. terebinth. may be used to accomplish the cure. But, notwithstanding these remedies, aided by antiphlogistic regimen, flooding often continues, and, by repeated detractions, gradually destroys the patient's health. In such a case, we must empty the uterus, as the only permanent remedy against future hæmorrhage.

There is, perhaps, no circumstance attendant on parturition, which exposes the patient to such imminent danger, as hæmorrhage at the commencement of labour; and, until the time of Dr. Rigby, it was not decided whether we should endeavour to restrain the discharge, and leave Nature to effect delivery, or introduce our hand into the uterus and bring away the child. This diversity of sentiment evidently arose from confounding the different causes of the disease with each other, and both proposals may be safely adhered to, when the phenomena that gave rise to them present themselves. Thus, when the placenta is partially detached, from some accidental cause, and the hæmorrhage is not so violent as to endanger life, we may wait, and have recourse to the means that are usually employed to restrain it; but, when the separation of the placenta arises from its position, as when it is attached to the cervix or os uteri, every return of pain must induce a return of bleeding, and the more the uterus dilates, the greater the discharge becomes, so that we cannot hesitate to effect delivery by turning the child. Dr. Rigby has not only proved the necessity of turning in such cases, but he has established the propriety of rupturing the membranes when flooding appears before delivery, and the placenta cannot be felt. Of 64

cases of accidental hæmorrhage, (hæmorrhage arising from *accidental*, and not necessary placental detachment) treated in this way, every patient was saved.

As hæmorrhage, occurring between the birth of the child and the delivery of the placenta, depends upon the retention of this body, its most effectual cure will be found in its removal. When the after-birth was not discharged within four hours after delivery, Denman and Hunter were accustomed to interfere; and other practitioners have been guided, not by the lapse of any specific period, but by the degree of pain, and the position of the placenta in the uterine cavity. After it has been withdrawn, every thing should be done to ensure firm and permanent uterine contraction. The patient must remain quiet, a bandage is to be applied around the region of the uterus, and every stimulant is to be avoided. But, notwithstanding every precaution, the womb may suddenly relax, an irregular inefficient contraction may have taken place, and a profuse hæmorrhage arise. In such an instance, attended with much exhaustion, we must exhibit some stimulant, as brandy or rum, apply cold to the pudenda, loins, and abdomen, grasp the uterus externally, or rub the integuments over it forcibly with the hand; and Dr. Gooch advises to introduce the left hand, closed, within the uterus, applying the right hand, open, to the outside of the abdomen, and then, between the two, compress the parts where the placenta was attached, and from which, chiefly, the blood is flowing. The following extract from one of Dr. Gooch's papers deserves insertion in this place:—

"I could easily understand that a contraction of the uterus, which would preclude hæmorrhage in the ordinary state of the circulation, might be insufficient to prevent it during violent action of the bloodvessels; and the inference I drew was, that in cases of hæmorrhage, dependent not on want of contraction of the uterus, but on the want of tranquillity of the circulation, a mode of treatment which would produce a cool skin and a quiet pulse, would be the best for preventing a recurrence of these floodings. How often a disturbance of circulation plays an important part in uterine hæmorrhage it is difficult for an individual to know; but I suspect sufficiently often to deserve the especial attention of practitioners. I advise them, when they meet with patients subject to hæmorrhage after delivery, to notice the state of the circulation before labour, and if disturbed, to employ means for tranquillising it before labour comes on. I advise them during labour, to use cordials cautiously, lest the placenta should separate during an excited state of circulation. I advise them, after delivery, though the uterus may feel contracted, to be slow to leave their patient, if the circulation be greatly disturbed." 447.

The great majority of affections occurring in the puerperal state, our author ascribes, either to the shock of delivery, to loss of blood before or after delivery, or to intestinal irritation. The last is, perhaps, occasionally a consequence, and not a cause; for, while intestinal accumulation before labour does produce injurious effects, it is certain that a predisposition to intestinal disorder does sometimes exist after confinement, which may be excited into activity by articles of diet, that at other times would be productive of no inconvenience. As we cannot, however, enter upon the consideration of each of these affections, we will confine our observations to one of the most interesting—*puerperal peritonitis.*

It may occur within 24 or 36 hours after delivery, in an acute or insidious form, and is accompanied by frequent pulse, rigors, heat of skin, pain of head, sickness and vomiting, oppression of the lochia, and mammary secretion. An attentive eye will also be struck with an expression of extreme pain and anxiety in the countenance, more especially on pressure. During its progress, if treatment have proved unavailing, the respiration becomes hurried, the abdomen prominent, the pulse increases in frequency and weakness, and the countenance wears a sunken aspect; but, if the patient is to recover, instead of this exasperation of symptoms, the pain will gradually abate, the pulse will become fuller and less quick, the respiration more easy, and an abundant lochial discharge, followed by secretion of milk, will not unfrequently crown the preceding salutary appearances.

"The treatment of puerperal inflammation within the abdomen is very simple, and does not admit even of medical controversy. The *first and great remedy* is the early abstraction of blood by the lancet; and on its bold, yet judicious repetition, will almost entirely depend the recovery of the patient: other remedies are valuable, but they are only subsidiary; after bleeding, they may and do aid in completing the cure, but without the previous abstraction of blood, they are almost entirely useless. 'The lancet,' says Burns, 'is the anchor of hope; it may indeed be pushed too far; it may be used by young practitioners, in cases of spasm, mistaken for peritonitis; but the error is safer than the contrary extreme; for, of the two evils, debility is more easily removed than inflammation. If the patient bear bleeding ill, and is not relieved, when it is first used, I apprehend that the case has not been simple peritoneal inflammation, but puerperal fever. If she bear it well, and the pulse becomes slower and fuller, and the pain abates, we are encouraged to repeat it.' As to the repetition of bleeding, and the manner of conducting it, I think it most important to remark, not only in reference to this, but to all puerperal diseases, that the mode proposed by Dr. Hall, *to place the patient upright, and to bleed to incipient syncope, is one of extreme value,* affording at once, perhaps, the safest rule, and the best diagnostic in these cases. If the affection be inflammation, much blood will flow before syncope will occur; and inversely, if much blood has flowed, the case is, most probably, decidedly inflammatory. In intestinal fever or irritation, on the contrary, early syncope from venesection is a certain, safe, and diagnostic event. In the first case, the patient will generally require to be soon visited and bled again; in the second, the attention must be fixed more particularly on other remedies, especially purgatives. There may, even after the fullest employment of general blood-letting, remain tenderness in the inflamed parts on pressure: and here, topical bleeding by leeches is peculiarly beneficial. The utility of purgatives requires no comment; in conjunction with bleeding, they are quite sufficient to subdue the disease. A difference of opinion prevails as to the efficacy of blisters: for myself, I should scarcely ever neglect their application, procuring the sleep which they may perhaps interrupt, by other means. It is scarcely necessary to caution the practitioner, that nothing beyond tea, gruel, or mild chicken broth, ought to be allowed during the existence of inflammation." 482.

We have now transferred to our pages a very copious specimen of Mr. Ashwell's volume, which concludes with an appendix, containing two papers from Dr. Blundell, "Researches, Physiological and Pathological," that we, in a former instance, fully noticed, "with the addition of three cases of the extirpation of the entire uterus," which we purpose commenting upon in a more specific form. As this work contains little novelty, either in the matter it communicates, or in the style of its execution, our task has been almost purely analytical, and our object, more to lay before our readers the obstetrical principles and practice of the present day, than to develop the merits of a performance, which can only be regarded as an enlarged manual of midwifery. Its style is generally rather loose, and occasionally inaccurate; its arrangement is, in many instances, confused; and tautology is, in some passages, perceptible. The author draws largely from authority, and extracts with judgment; his principles are generally correct, and his practice judicious; points of difficulty are seldom canvassed; and his aim, throughout, is evidently to teach the leading truths of his science with practical fidelity, without wishing to enhance himself by a parade of learning, or to bewilder his reader amid the doubts and speculations of obstetrical refinement.

